# Biomimetic nanoprobe-augmented triple therapy with photothermal, sonodynamic and checkpoint blockade inhibits tumor growth and metastasis

**DOI:** 10.1186/s12951-022-01287-y

**Published:** 2022-02-15

**Authors:** Xiaohong Lin, Tao He, Rui Tang, Qianru Li, Nianhong Wu, Yin Zhou, Hongye He, Li Wan, Ju Huang, Qinqin Jiang, Yixin Zhong, Zhuoyan Xie, Zhongqian Hu, Yang Zhou, Pan Li

**Affiliations:** 1 Institute of Ultrasound Imaging & Department of Ultrasound, The Second Affiliated Hospital of Chongqing Medical University, Chongqing Key Laboratory of Ultrasound Molecular Imaging, Chongqing, 400010 People’s Republic of China; 2 Department of Ultrasound, Chongqing General Hospital, Chongqing, 401147 People’s Republic of China; 3 Department of Orthopaedics, The First Affiliated Hospital of Chongqing Medical University, Chongqing, 400016 People’s Republic of China; 4 Department of Ultrasound, Zhongda Hospital, Medical School, Southeast University, Nanjing, 210009 People’s Republic of China; 5 Department of Ultrasound, The Third People’s Hospital of Chengdu City, The Affiliated Hospital of Southwest Jiaotong University, Chengdu, 610031 People’s Republic of China

**Keywords:** Immunotherapy, Photothermal therapy, Sonodynamic therapy, Cancer cell membrane, Multimodal imaging

## Abstract

**Background:**

Comprehensive antitumor therapy through integrated multimodal means has drawn increasing attention owing to its high efficiency and metastasis suppression.

**Results:**

We describe a synergistic triple protocol combining photothermal and sonodynamic therapy (PTT and SDT), together with immune checkpoint blockade for the inhibition of breast cancer growth and metastases in the 4T1 mouse model. PTT and SDT are synergistically augmented by a novel multimodal imaging nanoprobe integrated with cancer cell membrane-biomimetic nanoparticles (CHINPs) loaded with superparamagnetic iron oxide (SPIO) and hematoporphyrin monomethyl ether (HMME). CHINPs exhibit excellent homologous tumor targeting, and are sequentially triggered by ultrasound and near infrared (NIR) light under the guidance of magnetic resonance, photoacoustic and photothermal imaging, leading to complete in situ tumor eradication and systemic anti-tumor immune activation. Further combination of this approach with immune checkpoint blockade therapy is shown to suppress tumor metastasis.

**Conclusion:**

This work provides proof-of-principle for triple therapy using multimodal imaging-guided PTT/SDT based on biomimetic nanoprobes in combination with immunotherapy to eliminate tumors.

**Graphical Abstract:**

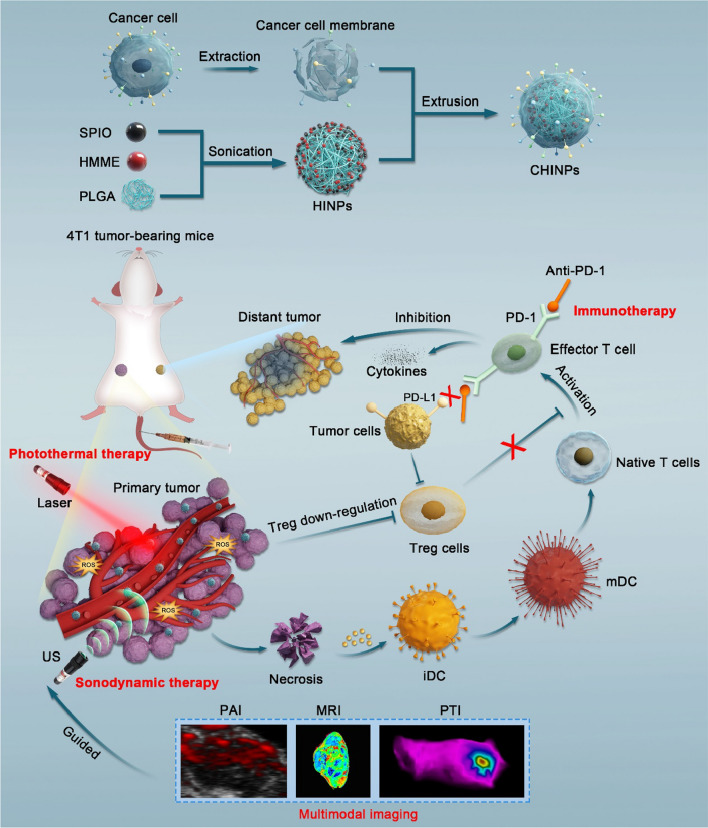

**Supplementary Information:**

The online version contains supplementary material available at 10.1186/s12951-022-01287-y.

## Background

The occurrence and development of tumors is complex and currently no single treatment method entirely meets clinical needs [1]. Therefore, combined anti-tumor therapy strategies exploring different therapeutic approaches and/or related mechanisms of action have great application prospects. Showing both effectiveness and low toxicity, numerous studies have now highlighted the potential of comprehensive antitumor therapy with multifunctional nanoplatforms to solve many current limitations of cancer treatment [2–5]. With the rapid development of nanomolecular targeted therapy [6], a number of minimally invasive or non-invasive therapies have emerged, which are safe and controllable [7–11]. Among them, sonodynamic therapy (SDT) is a non-invasive therapeutic strategy for the treatment of deep tumors. It uses ultrasound to activate a sonosensitizer that produces reactive oxygen species (ROS), causing apoptosis and necrosis in tumor cells [11–18]. The non-invasiveness nature of SDT, its low cost and high tissue penetration depth, has increasingly attracted more attention from clinicians and scientists [19, 20]. However, the severe hypoxia in the solid tumor microenvironment (TME) and oxygen consumption during SDT greatly hinders the ROS production, which leads to dramatic decline of SDT therapeutic efficiency [21–23].

Photothermal therapy (PTT) represents another promising anti-tumor technology with great clinical application potential. It utilizes photothermal transduction agents (PTAs) to assimilate the energy from light and convert the energy into heat which is commonly known as photothermal effect, leading to the hyperthermia and tissue ablation in the tumor region. However, the biggest problem of PTT is the limited depth of light penetration, which may cause incomplete ablation of tumors outside the scope of irradiation [24], therefore PTT is only effective for superficial lesions. Although near-infrared (NIR) light has improved the depth of penetration compared to visible light, it is still challenging to transform this technology to clinical practice due to the limited tissue absorption and light scattering [25].

In recent years, the combination of PTT and SDT offers much promise for synergistic antitumor therapy. On the one hand, the light modality has good sensitivity with adjustable dosage that allows precise targeting of tumors; more notably, the resulting thermal effect can improve SDT by enhancing blood flow and oxygenation of the tumor [12]. On the other hand, the deep tissue penetration of SDT can overcome the inherent deficiency of PTT in targeting deeper tumors. Therefore, the photothermal-sonodynamic combined therapy has achieved significant synergistic therapeutic effects [10–12, 26–28]. However, tumor metastasis remains an intractable problem even if the primary tumor is locally destroyed by PTT or SDT.

Fortunately, accumulating evidence has revealed that PTT or SDT can not only directly kill tumor cells, but result in tumor fragments to release antigens serving as an in situ vaccine, activating specific antitumor immune responses [29–34]. This rationale has been demonstrated in many preclinical animal models [35–37] and validated in preliminary clinical trials in patients with breast cancer [38–40]. However, the "abscopal effect" induced by PTT or SDT is not capable of bringing about effective immunotherapy to prevent tumor growth and metastasis. Besides, current PTAs and sonosensitizers are mainly prepared with exogenous materials which suffer low bioavailability, poor targeting ability and short circulation time in vivo due to the phagocytosis of reticuloendothelial system (RES) [41–52], together providing major obstacles for the broad clinical application of PTT or SDT.

Cell membranes coated biomimetic nanotechnology is a new emerging technology in recent years, which was first published in 2011 in PNAS by Zhang Lab [53]. Cancer cell membranes (CCMs) contain multiple endogenous protein and lipid bilayer layers, the membrane protein molecules including CD47 on the surface of breast cancer cells endow nanoparticles with the property of immune tolerance and prevent the macrophage uptake, which are conducive to improve the biocompatibility of nanoparticles and prolong their circulation time in vivo [48, 54]. Furthermore, specific protein molecules on CCMs enable nanoparticles to actively target homologous tumors [48, 52, 55–60].

Tumor immunotherapy, which induces the innate immune system to kill tumor cells, has provided a promising new avenue for cancer control. PD-1 checkpoint blockade immunotherapy has shown promise in various malignancies with notable clinical efficacy, long lasting response, and low toxicity. However, the low response rate, tumor resistance immune-related adverse events [61–64] limit its wide clinical use. Combination therapy can be an effective tactic to induce more mature dendritic cells (DCs) and increase the content of effector T cells, which are the main approaches to greatly improve the performance of PD-1 cancer immunotherapy [65–67]. Moreover, uniting local ablation and immunotherapies such as PD-1 inhibitors is one of the most potent regimens that oncologists can manipulate [68, 69].

**Scheme 1:**
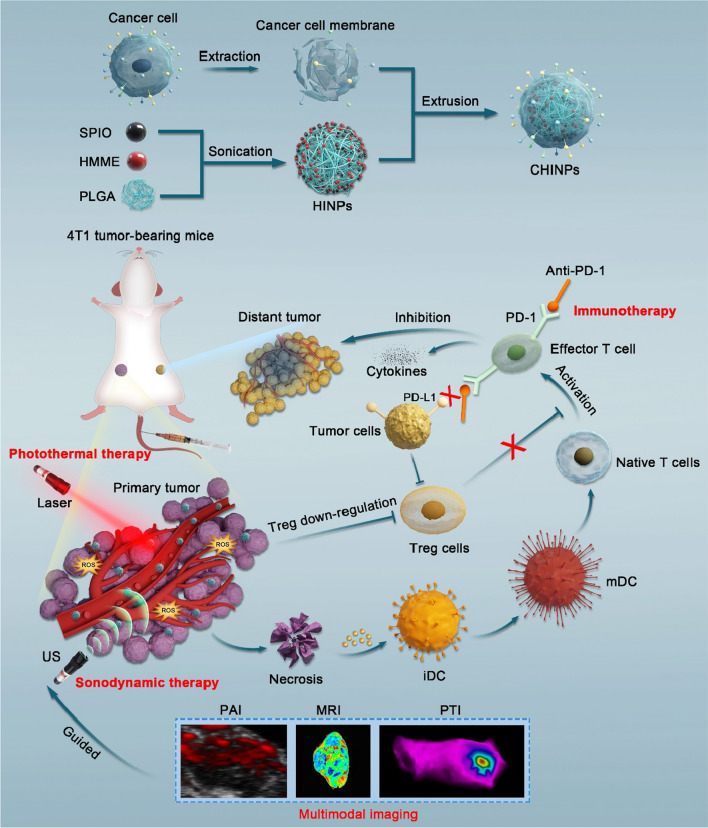
Schematic illustration of the synthesis of CHINPs and the combined effects of PTT/SDT enhanced anti-PD-1 against primary and distant tumors. Preparation of HINPs based on a simple double-emulsion approach, Cancer cell membranes were further modified onto the surfaces of HINPs to construct biomimetic Nanoparticles. CHINPs actively targets homologous tumors, eliminating primary tumors with PTT/SDT under the guidance of MRI /PAI/ PTI multimode imaging, exposing tumor associated antigens and inducing systemic immunity through increased CD8^+^ T cells and decreased Treg cells. Anti-PD-1 blocks the PD-1/PD-L1 checkpoint and further enhances T cells to attack tumor cells

In this study, we developed a combined triple therapeutic strategy, which integrated PTT, SDT and anti-PD-1 immunotherapy under multimodal imaging guidance to eliminate 4T1 tumors in mice (Scheme 1). PTT and SDT were employed to synergistically destroy the primary tumor and simultaneously activate systemic immune responses, effectively improving treatment outcomes of PD-1 checkpoint blocking antibodies and inhibiting tumor metastasis. The PTT/SDT regimen was augmented by 4T1 cancer cell membranes modified polylactic glycolic acid (PLGA) nanoparticles loaded with a US responsive sonosensitizer, hematoporphyrin monomethyl ether (HMME) and a laser responsive PTA, superparamagnetic iron oxide (SPIO). The CHINPs showed homologous tumor targeting property and enhanced magnetic resonance, photoacoustic and photothermal imaging during the treatment. Therefore, our work develops a multimodal imaging-guided triple therapeutic nanoplatform, which proposes a novel strategy to eradicate tumors.

## Materials and methods

### Materials

Carboxy-terminated polylactic acid/glycolic acid PLGA (PLGA-COOH polymerization ratio: 50:50, molecular weight: 12,000 Da) was purchased from Jinan Daigang Biotechnology Co., Ltd. (Jinan, China). Hematoporphyrin monomethyl ether (HMME) was purchased from Shanghai DB Chemical Technology Co., Ltd. (Shanghai, China). Oleic-acid-modified superparamagnetic iron oxide (SPIO) nanoparticles (d = 10 nm) were purchased from Ocean Nano Tech, Inc., (AR, USA). The cell membrane protein and cytoplasmic protein extraction kit and phenylmethanesulfonyl fluoride (PMSF) were purchased from Beyotime (Shanghai, China). Calcein-AM, PI and CCK-8 assay kits were purchased from Dojindo Laboratories (Kumamoto, Japan). Anti-mouse PD-1 (CD279, Lot: 78012ON, Catalog No. BE0146) was obtained from Bioxcell (USA). ELISA kits were purchased from Elabscience Biotechnology Co., Ltd (Wuhan China). Antibodies to cell surface markers for flow cytometry analysis were purchased from BioLegend, Inc., (CA, USA). All unspecified reagents used were of analytical grade or better.

### Cell culture and tumor model

Five types of cells including 4T1, MDA-MB-231, MG63, B16F10 and RAW264.7 were routinely cultured in RPMI-1640 medium supplemented with 10% FBS (v/v) and 1% penicillin–streptomycin (v/v) under standard conditions (5% CO_2_, 37 ℃).

Female Balb/c mice and Kunming mice (6–8 weeks old, 16–20 g weight) were obtained from the Laboratory Animal Center, Chongqing Medical University. All animal experiments were approved by the Animal Protection Committee of Chongqing Medical University (approval number: CQLA-2018-0505). The orthotopic 4T1 tumor-bearing model used for imaging purposes was established by injecting 4T1 cells (1 × 10^7^) dispersed in 100 µL PBS into the fat pad of right fifth mammary gland in each mouse (Unilateral model). In addition, a bilateral model was established by injecting 4T1 cells (2 × 10^6^) dispersed in 100 µL PBS into the fat pad of the left fifth mammary gland to simulate a distant metastatic tumor when the tumor on the right side reached 50–60 mm^3^ (6 days later). According to the standard animal protocol, mice with tumors greater than 1000 mm^3^ were euthanized.

### Synthesis of HINPs

The nanoparticles (HINPs) encapsulating HMME and SPIO were fabricated by using a typical double-emulsion process (w/o/w) [32, 70]. Briefly, 1.4 mg HMME and 1.05 mg SPIO were added into 2 mL of PLGA (50 mg) dissolved in dichloromethane (oil phase), and 200 μL double distilled water (water phase) was added. Then, the mixture was emulsified by using an ultrasonic probe (Sonics & Materials, Inc., USA) with power of 60 W for 3 min to form the first w/o emulsion. The w/o emulsion was then poured into 10 mL 4% w/v PVA solution and emulsified for 3 min at the same power for the second w/o/w emulsion. Subsequently, 20 mL 2% v/v of isopropyl alcohol solution was added to the above emulsion to evaporate organic solvent for 6 h at room temperature. Lastly, the HINPs were collected after centrifugation at 10,000×*g* for 10 min. The DIR-labeled HINPs were prepared by the same method. The encapsulation efficiency (EE) and loading capacity (LC) of HMME and SPIO in HINPs were evaluated by UV–vis spectra technology (UV-3600, Shimadzu, Japan) and inductively coupled plasma mass spectrometry (ICP-MS) (Agilent ICPMS 7700), respectively. The EE and LC were calculated as described below (n = 3):

$${\text{EE }}\left( \% \right) \, = \frac{{\text{HMME or SPIO encapsulated in nanoparticles}}}{{\text{Total HMME or SPIO added}}} \times {1}00\%$$

$${\text{LC }}\left( \% \right) = \frac{{\text{HMME or SPIO encapsulated in nanoparticles}}}{{\text{Weight of nanoparticles}}} \times {1}00\%$$

### Cancer cell membranes (CCMs) extraction

4T1 cell membranes were extracted using the Membrane Protein Extraction Kit according to the instructions provided by Beyotime Biotechnology. Briefly, 4T1 cells were incubated in cell culture dishes with diameter of 15 cm, and then the cells (1 × 10^8^) were collected by a cell scraper and centrifuged at 700×*g* for 5 min. The cell precipitation was resuspended in precooled PBS buffer (pH = 7.4) followed by centrifugation at 600×*g* for 5 min. In order to remove the residual PBS buffer, further centrifugation for 1 min was performed. The obtained cell pellets were suspended in Membrane Protein Extraction solution (3 mL), and phenylmethanesulfonyl fluoride (PMSF, 1 × 10^–3^ M) was added. After that, the cells were incubated in an ice bath for 15 min. Thereafter, repeated freeze-thawing was carried out to break the cells in the above solution and then centrifuged at 700×*g* for 10 min at 4 ℃. The collected supernatant was further centrifuged at 14,000×*g* for 30 min to collect the CCMs. The CCMs was collected and lyophilized for later use [48, 57]. All cell membrane extraction processes were carried out in an ice bath.

### Preparation and characterization of CHINPs

CHINPs were prepared according to the methods reported in the previous literature [48, 52, 55]. CCMs was mixed with HINPs under eddy agitation, and the mixture was extruded through a 400 nm polycarbonate membrane for at least 5 cycles (Avestin mini extruder, Canada). Finally, the excess cell membrane was removed by centrifugation at 4 ℃ (10,000×*g*, 10 min) for two times to obtain CHINPs. CHINPs morphology was observed under the Cy5 channel of laser confocal microscope (CLSM, LSM710, Carl Zeiss, Germany), and structurally analyzed by transmission electron microscope (TEM Hitachi H-7600, Japan). The UV–Vis spectrum of PLGA, HMME, SPIO or CHINPs was then detected. The Fe content in CHINPs was detected by ICP-MS and X-ray diffraction (XRD) system (Bruker D8 Advance). The colloidal stability of CHINPs in PBS, water containing 1640 cell cultured medium, 10% or 20%FBS were evaluated at 1, 3, 5, 7 d after incubation. The particle size and zeta potential of CCMs, HINPs or CHINPs were measured with a dynamic laser scattering (DLS) particle sizer (ZEN3600, Malvern Instruments, UK). The membrane proteins carried by nanoparticles were detected by protein gel electrophoresis (SDS-PAGE). The integrity of the cell membrane-coated HINPs was determined by the co-localization of the membrane and cores. We labeled the cell membrane with DIO (3,3′-dioctadecyloxacarbocyanine perchlorate) a green fluorescent dye and then co-extruded it with HINPs to obtain CHINPs. Then the CHINPs were observed under the Cy5 and FITC channel of CLSM.

### In vitro targeting of CHINPs to homotypic cells

To determine the in vitro targeting effects of CHINPs, 4T1 cells were seeded in the confocal dishes (1 × 10^5^ cells per well) and incubated under the condition of 5% CO_2_ at 37 °C. 24 h later, the cells were then treated with HINPs or CHINPs suspensions (equivalent PLGA concentration: 1.0 mg mL^−1^) for 1, 2, 3, 4 h. Subsequently, the cells were washed by PBS and fixed in 4% paraformaldehyde for 30 min. Then DAPI was added to stain the cell nuclei and the dishes were observed under CLSM. In addition, 4T1 cells were seeded into 6-well plates and treated with HINPs or CHINPs for 1, 2, 3, 4 h, respectively. Then the cells were collected after trypsinization, and re-suspended in PBS to detect the fluorescence intensity by Flow cytometry (Cytoflex, USA).

In order to prove our assumption that CCMs coating would be self-recognized by homotypic cancer cells, the cell internalization of CHINPs and HINPs were evaluated in five different cell lines including 4T1 cells, MDA-MB-231cells, MG63 cells, B16F10 cells and RAW264.7 cells upon 4 h co-incubation. Then DAPI were added to stain the nuclei for 10 min. The uptake of nanoparticles were observed under CLSM and quantitatively analyzed by Flow cytometry.

### In vivo biodistribution of CHINPs

4T1 tumor-bearing mouse (unilateral tumor) were prepared, 200 µL HINPs or CHINPs (equivalent PLGA concentration: 10 mg mL^−1^) were injected through tail vein (3 per group). Fluorescence imaging (Xenogen IVIS Spectrum, PerkinElmer, USA) was then performed at different time points (pre, 1 h, 3 h, 6 h, 24 h, 48 h) and the fluorescence intensity in tumor regions at each time point was quantitatively analyzed by Living Image 4.5 software. Another six mice were sacrificed 24 h after injection with HINPs or CHINPs (3 per group), major organs (heart, liver, spleen, lung, kidney) and tumors assessed by fluorescence imaging. Thereafter, tumor tissues were collected for Prussian blue staining.

To quantitatively analyze the accumulation of nanoparticles in tumor tissue, nine 4T1 tumor-bearing mice were randomly divided into three groups (n = 3), and 200 µL PBS, CHINPs or HINPs (equivalent PLGA concentration: 10 mg mL^−1^) were injected via tail vein, respectively. All mice were sacrificed at 24 h after injection, the main organs and tumors were weighed and dissolved in 10 mL nitric acid. Then the Fe content in these samples was measured by ICP-MS.

### Detection of ROS levels

Reactive oxygen species (ROS) levels were detected by the Singlet Oxygen Sensor Green (SOSG) fluorescence probe. Briefly, 0.3 mL SOSG dissolved in methanol was added to CHINPs suspensions (20 μg/mL). The mixture was activated by low-intensity focused ultrasound (LIFU, LM.SC051 ACA; Institute of Ultrasound Imaging, Chongqing Medical University, China) for different durations (0 s, 15 s, 30 s, 60 s, 90 s, 120 s, 150 s, respectively). The irradiation parameters were set as follows: 2.0 W cm^−2^, 1 MHz, 50% duty cycle. Under the US irradiation, SOSG emit fluorescence at 525 nm after capturing ^1^O2. And the fluorescence intensity was recorded by fluorescence spectrophotometer.

Additionally, cellular ROS levels were determined by 2',7'-dichlorofluorescein diacetate (DCFH-DA). 4T1 cells seeded in the confocal dishes (1 × 10^5^ cells per well) were randomly divided into nine groups including: (i) Control group, (ii) Laser group, (iii) US group, (iv) Laser + US group, (v) CHINPs group, (vi) CHINPs + Laser group, (vii) CHINPs + US group, (viii) HINPs + Laser + US group and (ix) CHINPs + Laser + US group. The cells in various groups were treated with different nanoparticles for 4 h. Then, the cells were further incubated with DCFH-DA (1 × 10^–5^ M) for 30 min before US (1.0 MHz, 2.0 W cm^−2^, 50% duty cycle, 2 min) or 808 nm laser (2 W cm^−2^, 10 min) irradiation. After another 1 h of incubation, the cells were washed three times with PBS and observed under CLSM. In parallel, flow cytometry was used to detect cellular ROS levels.

### In vitro cytotoxicity measurements

For in vitro cytotoxicity assay, 4T1 cells were seeded in 96-well plates at the density of 3 × 10^3^ cells and allowed to attach for 24 h. Then the cells were treated with fresh medium containing different concentrations (equivalent PLGA concentration 1, 2, 3, 4, 5 mg mL^−1^) of CHINPs. After incubation for desired time duration (i.e., 3 h, 6 h 12 h and 24 h), the cell viabilities were tested via a typical CCK-8 assay.

### In vitro PTT/SDT-induced cytotoxicity assays

4T1 cells were seeded in 6-well plates at the density of 1 × 10^5^ cells for 24 h, nine different of PTT/SDT treatments were conducted on the cells as detailed in the ROS experiments. Briefly, the cells treated with 100 µL CHINPs or HINPs (equivalent PLGA concentration: 5 mg mL^−1^) for 4 h before giving the various treatments (Laser: 2 W cm^−2^, 10 min; US: 1.0 MHz, 2.0 W cm^−2^, 50% duty cycle, 2 min). Thereafter, the cells were stained with Annexin V FITC/PI and the proportions of apoptosis in each group were analyzed by flow cytometry.

Furthermore, cell viabilities were also visualized by calcein-AM/PI staining. 4T1 cells were seeded in the confocal dishes (1 × 10^5^ cells per well) for 24 h and cocultured with different nanoparticles for 4 h. The Calcein-AM (10 μL) and PI (20 μL) dispersed in PBS (10 mL) were used to replace the cell culture media and stained live (green) and dead (red) cells after varied treatments as mentioned above. After 15 min staining, the cells were washed by PBS for three times and observed by CLSM.

### In vitro and in vivo photothermal performance

To test the in vitro photothermal performance of nanoparticles, 100 µL CHINPs at different concentrations (equivalent PLGA concentrations at 0.625, 1.25, 2.5, 5 and 10 mg mL^−1^) in a 96-well plate were irradiated by an 808 nm laser (2.0 W cm^−2^, 10 min). An infrared camera (Fotric 226, Shanghai, China) was used, and the temperatures were recorded in real-time. CHINPs at a concentration of 10 mg mL^−1^ (equivalent PLGA concentrations) were irradiated at various irradiation intensities (0.5, 1.0, 1.5, 2.0, and 2.5 W cm^−2^) for 10 min. To examine the photostability, a CHINPs suspension was exposed to 808 nm laser irradiation until the temperature increased to 48 °C. Subsequently, the suspension was cooled to ambient temperature by turning off the 808 nm laser. The laser on and off procedure was repeated for 6 cycles, and the suspension temperature was recorded in real time using a thermal infrared camera during all irradiation processes.

4T1 tumor-bearing mice (Unilateral model) were also used to evaluate PTI performance of nanoparticles in vivo. The mice were randomly divided into three groups (n = 3), and were injected with 200 µL PBS, CHINPs or HINPs (equivalent PLGA concentration: 10 mg mL^−1^) via tail vein, respectively. PTI images were obtained 24 h post injection under 808 nm laser irradiation (2 W cm^−2^, 10 min). The mice were then sacrificed 24 h after treatment and the tumors were collected for H&E staining to evaluate the histological changes.

### In vitro and in vivo MRI performance

CHINPs suspension at different concentrations (Fe concentrations: 0.026, 0.052, 0.104, 0.208, 0.416, 0.832 and 1.664 mM) were prepared and added into 2 mL Eppendorf tubes, respectively, and the in vitro properties of nanoparticles were detected using an MRI system (Siemens Prisma, 3.0T, The Second Affiliated Hospital of Chongqing Medical University) with a gradient echo sequence, and the corresponding T_2_ relaxation time was analyzed and processed by Syngo Via software.

4T1 tumor-bearing mice (Unilateral model) were injected with 200 µL HINPs or CHINPs (3 per group; equivalent PLGA concentration at 10 mg mL^−1^) through the tail vein and T2-weighted MR imaging was performed. The MRI signals of the tumor area were captured at different time points (pre, 1 h, 3 h, 6 h, 24 h, 48 h) under a special coil for small animals. MRI parameters were set as follows: T2WI: (repetition time (TR)/echo time (TE): 3200/96 ms, thick: 1.4 mm, FOV: 120 × 100 mm. T2MAP: (repetition time (TR)/echo time (TE): 2390/15 ms, thick: 1.4 mm, FOV: 85 × 85 mm). The acquired data were analyzed and processed by Syngo Via software.

### In vitro and in vivo PAI performance

CHNPs, CINPs and CHINPs suspensions at different concentrations (equivalent PLGA concentration: 2, 4, 6, 8, 10 mg mL^−1^) were prepared and added into different gel pores. PBS was used as the control group, the photoacoustic imaging of nanoparticles in vitro was obtained with the Vevo LAZR photoacoustic imaging system (Visual Sonics Inc., Toronto, Canada), and the intensity of the photoacoustic signal was quantitatively analyzed with the corresponding system software.

4T1 tumor-bearing mice (Unilateral model) were injected through the tail vein with HINPs or CHINPs (3 per group; equivalent PLGA concentration at 10 mg mL^−1^, 200 µL) and photoacoustic imaging of the tumor region performed at different time points (pre, 1 h, 3 h, 6 h, 24 h, 48 h) to evaluate the photoacoustic imaging performance in vivo. PAI parameter was set as follows: PAI gain: 45 dB; Focus depth: 10 mm.

### In vivo SDT/PTT synergistic anti-PD-1 immunotherapy

4T1 tumor-bearing mice (bilateral tumor model) were randomly divided into 9 groups (n = 6) including: (i) Control, (ii) Laser + US, (iii) CHINPs, (iv) anti-PD-1, (v) CHINPs + Laser, (vi) CHINPs + US, (vii) HINPs + Laser + US, (viii) CHINPs + Laser + US, (ix) CHINPs + Laser + US + anti-PD-1. The mice were intravenously injected with 200 µL PBS, HINPs or CHINPs suspension (equivalent PLGA concentration: 10 mg mL^−1^). The primary tumor was irradiated with an 808 nm laser (2 W cm^−2^, 10 min) and/or US (1.0 MHz, 2 W cm^−2^, 50% duty cycle, 5 min) after 24 h post injection. Anti-PD-1 antibodies at the dose of 50 μg /mouse were administered on days 1, 4, 7 and 10. Animal weight and tumor volume were measured every other day for 16 days. The tumor volume was calculated using the formula as follows: V = (length × width^2^)/2 mm^3^. At day 3 after treatment, one mouse in each group was sacrificed, and the primary tumor tissues were collected and stored in 4% formaldehyde with H&E, TUNEL and Ki67 staining performed to observe proliferation and apoptosis.

### Immune status investigations

To investigate the in vivo anti-tumor immune responses against mimic distant tumors, the 4T1 tumor-bearing mice received above were sacrificed on day 7 post treatment, the distant tumors were harvested and produced a single-cell suspensions. The harvested cells were further stained with several antibodies: CD11c-FITC (#117306), CD80-PE (#104708) and CD86-APC (#105012); T cells with CD3-FITC (#100204), CD4-PE (#100408), CD8-APC (#100712) and FOXP3-Alexa Fluor® 647(#126408) and then analyzed by flow cytometry. In parallel, serum cytokines levels including TNF-α, IFN-γ, and IL-12, were analyzed using by ELISA (Wuhan servicebio technology CO., LTD).

### In vivo toxicity of CHINPs

Healthy Kunming mice (n = 25, 6–8 weeks old) were selected and five mice were used as control, and the other twenty mice were injected with 200 µL CHINPs (equivalent PLGA concentration at 10 mg mL^−1^). Treated mice were sacrificed at 1d, 7d, 14d and 28d after injection (5 per group), and control mice were sacrificed at 28d. The major organs (including the heart, liver, spleen, lungs, and kidneys) were collected for H&E staining to evaluate the histopathologic toxicity. Blood samples were collected for routine blood tests and biochemical examinations.

### Statistical analysis

All data were expressed as the means ± standard deviation (SD), and the significance of differences among groups was evaluated with either one-way ANOVA (multiple groups) or Student’s t-test (comparisons of two groups) (*p < 0.05, **p < 0.01, ***p < 0.001, **** < 0.0001).

## Results and discussion

### Synthesis and characterization of CHINPs

**Fig. 1:**
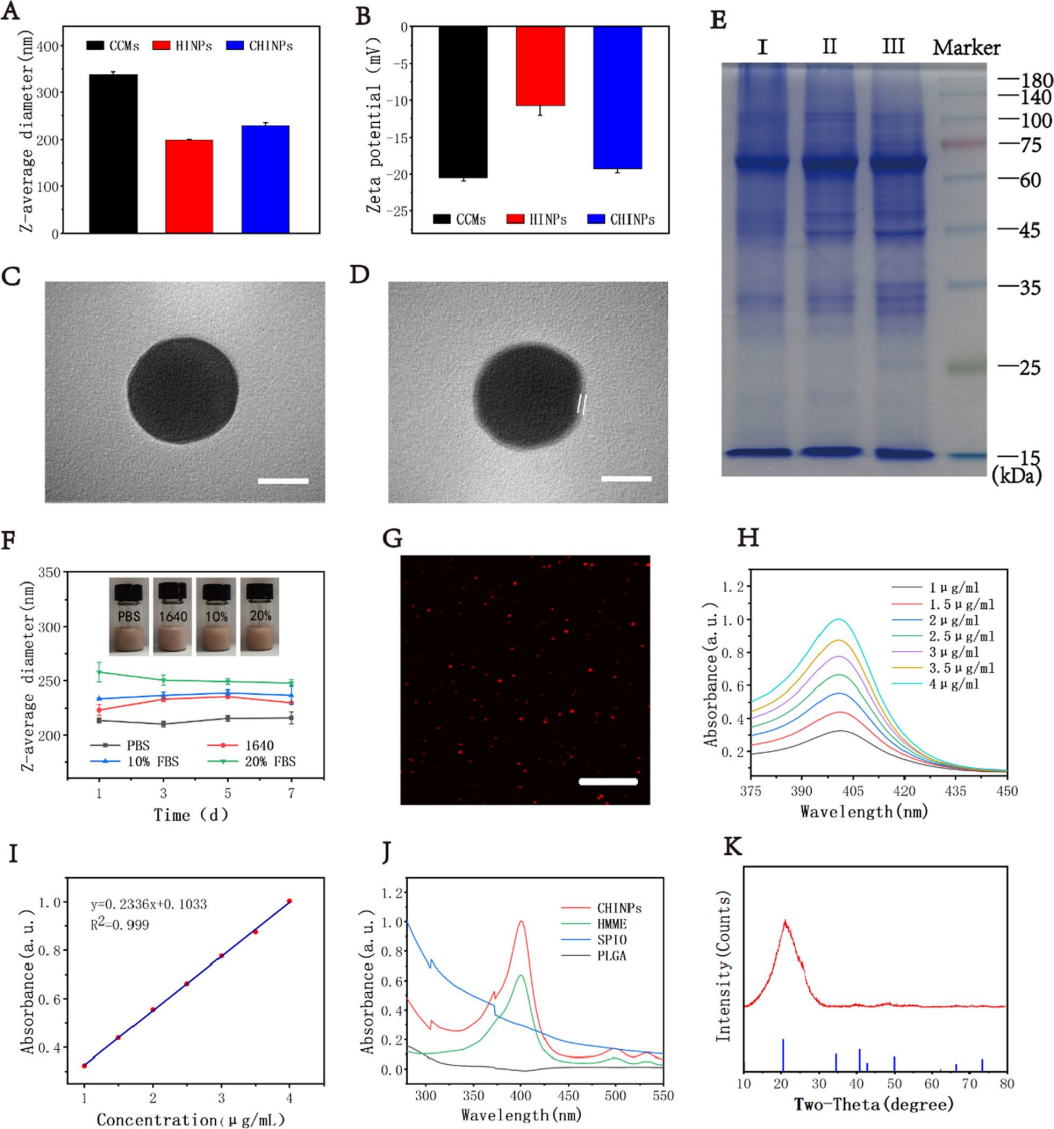
CHINPs characterization. **A** Z-average diameter and **B** zeta potentials of the CCMs, HINPs and CHINPs. **C** TEM image of HINPs and **D** CHINPs. Scale bar: 100 nm. **E** SDS-PAGE protein analysis of different samples. Samples were stained with Coomassie Blue. I): tumor cells lysate, II): CCMs, III): CHINPs. **F** The stability of CHINPs in PBS, water containing 1640 medium, 10% or 20% FBS. Inset: photographs of CHINPs after incubation in different medium. Data shown are mean ± SD (n = 3). **G** CLSM image of CHINPs, Scale bar: 25 µm. **H** UV–Vis spectrum of HMME and **I** Absorbance intensity of HMME at 401 nm. **J** UV–Vis spectrum of PLGA, HMME, SPIO and CHINPs. **K** XRD patterns of CHINPs

PLGA nanoplatforms were used to co-encapsulate two hydrophobic molecules including HMME and SPIO to produce HINPs. The EE and LC were controllable, which varied with the different initially feeding dose. (Additional file 1: Figs. S1–S6). The resulting HINPs formulation possessed a mean diameter of 198.93 ± 1.44 nm and a zeta-potential of − 10.74 ± 1.33 mV as determined by DLS, and the EE and LC are 82.12% and 3.16% for HMME, and 89.21% and 1.83% for SPIO, respectively. Cancer cell membranes (CCMs) extracted from 4T1 breast cancer cells and HINPs were further co-extruded to fabricate CHINPs by top-down assembly [48, 52, 55]. The average size of CHINPs increased to 228.07 ± 6.21 nm and the zeta-potential decreased to − 19.27 ± 0.55 mV, indicating that the nanoparticles had been successfully coated with CCMs (Fig. 1A, B). Under transmission electron microscopy (TEM) (Fig. 1C, D), CHINPs appeared as a typical "shell core" like spherical structure with 9 nm CCMs coating on the surface, which is consistent with previous reports [52, 56, 58, 59]. The DIO-labeled CCMs coating was also displayed by the green fluorescence from the shell of CHINPs (Additional file 1: Fig. S7). In addition, CHINPs and CCMs displayed nearly identical protein profiles as assessed by SDS-PAGE (Fig. 1E), which could further confirm the successful coating. CHINPs featured a favorable structural stability in a variety of media including PBS, water containing 1640 cell cultured medium, and 10% or 20% FBS (Fig. 1F), and no obvious aggregation or precipitation was found even 7 days later (Additional file 1: Fig. S8).

Since HMME has autofluorescence [71], CCMs-coated CHINPs nanoparticles showed marked red fluorescence under CLSM (Fig. 1G). HMME showed an absorption peak at 401 nm, and the absorbance value rose with the increasing drug concentration as assessed by UV spectrophotometry (Fig. 1H), which were linearly correlated (Fig. 1I). The characteristic absorption peak of HMME was displayed in CHINPs in UV absorption spectrum (Fig. 1J), which indicated that HMME had been successfully wrapped into the PLGA nanoparticles. Fe content was detected in CHINPs by ICP-MS and X-ray diffraction (XRD) (Fig. 1K), indicating that SPIO was successfully loaded into CHINPs and could be used as a MRI contrast agent [72, 73]. In summary, the above results suggest that a novel cancer cell membrane-coated nanoprobe (CHINPs) loaded with SPIO and HMME is successfully synthesized.

### In vitro targeting of CHINPs to homotypic cells

**Fig. 2:**
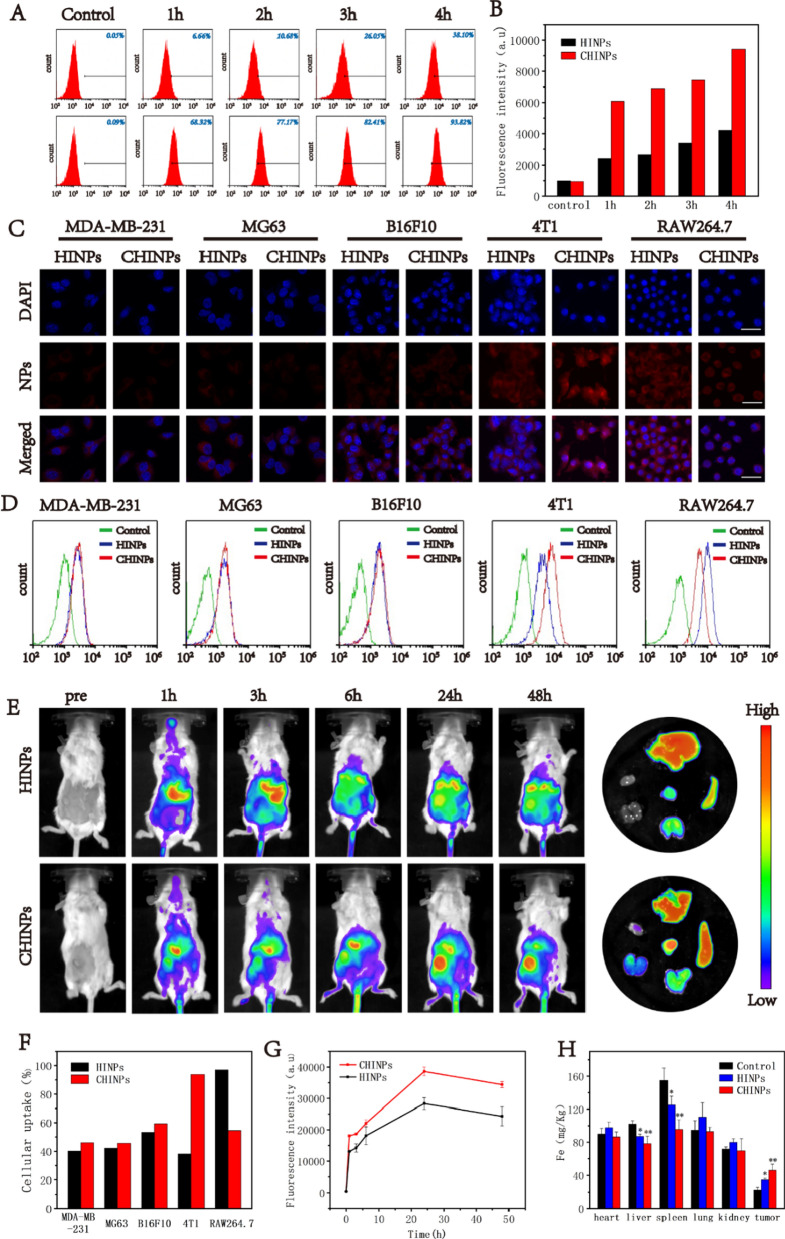
In vitro and in vivo homologous-targeted effects of CHINPs and biodistribution. **A** Flow cytometric profiles of 4T1 cells treated with HINPs and CHINPs and **B** the corresponding mean fluorescence intensity. **C** CLSM images of different cells treated with HINPs, CHINPs. Scale bar: 20 μm. **D** Flow cytometric profiles of different cells treated with HINPs, CHINPs and **F** the corresponding cellular uptake. **E** In vivo distribution of HINPs and CHINPs in 4T1 tumor-bearing Balb/c mice examined by fluorescence imaging at different time points and Ex vivo tissue distribution in the main organs: heart, liver, spleen, lung, kidney, and tumor. **G** The corresponding mean fluorescence intensity of tumor by in vivo fluorescence imaging. **H** Quantitative biodistribution of CHINPs in Balb/c mice determined by ICP-MS. Data shown are mean ± SD (n = 3). Statistical differences determined by one-way ANOVA; *p < 0.05, **p < 0.01

Taking advantage of red fluorescence from HMME, the uptake of nanoparticles by 4T1 cells could be clearly shown under CLSM. Significant stronger red fluorescence was shown in CHINPs compared to that in HNIPs at different time points (Additional file 1: Fig. S9), indicating that more CHINPs nanoparticles were taken in by 4T1 cells. And the cellular uptake was increased over the incubation time. The higher uptake of CHINPs was also verified by the fluorescence intensity from flow cytometry (Fig. 2A, B). These results suggest that the outer membrane endow CHINPs with the targeting ability to 4T1 cells.

After incubation for 4 h, the fluorescence intensity of HMME from CHINPs was much higher in 4T1 cells than that in heterotypic cells, suggesting the highly specific self-recognition affinity of CHINPs to the homologous cells. However, in RAW264.7 cells, compared with HINPs group, CHINPs group displayed weaker fluorescence (Fig. 2C, D, F), suggesting that CCMs coating could reduce the macrophage engulfment. These results demonstrated that the cell membrane coating played a critical role in cellular uptake of the CHINPs.

### In vivo homologous-targeted imaging and biodistribution of CHINPs

The homotypic cancer-targeting ability of CHINPs in vivo was evaluated by living fluorescence imaging system (IVIS) after i.v. administration with nanoparticles into 4T1 tumor bearing Balb/c mice. Fluorescence intensity of CHINPs in vitro was in a concentration-dependent manner (Additional file 1: Fig. S10). Nanoparticle distribution in tumor tissues was increased gradually over the time and reached a peak at 24 h after injection, and a marked accumulation was shown in tumor region in CHINPs group (Fig. 2E, G). More importantly, the accumulation of CHINPs in tumor region was significantly higher than that of HINPs, which was further confirmed by Prussian blue staining (Additional file 1: Fig. S11) where more remarkable iron particle deposition was observed in the tumor tissue in CHINPs group. The Fe content in the tumor tissue in CHINPs group was 1.4-fold higher than that in HINPs group as determined by ICP-MS (Fig. 2H), which was consistent with the in vivo imaging results. The accumulation of nanoparticles in liver and spleen were still seen due to the non-specific interception by RES [74, 75]. However, the uptake of CHINPs by RES could be significantly reduced after CCMs coating. The accumulation of CHINPs in liver and spleen was much lower than that of HINNPs (Fig. 2E, H). Overall, these data indicate that CHINPs are endowed with homologous targeting ability to 4T1 tumor by CCMs coating and accumulated in tumor site, thus can be used as a biomimetic nanoprobe for targeted breast cancer imaging and therapy.

### In vitro cytotoxicity and synergistic antitumor effects of PTT/SDT

**Fig. 3:**
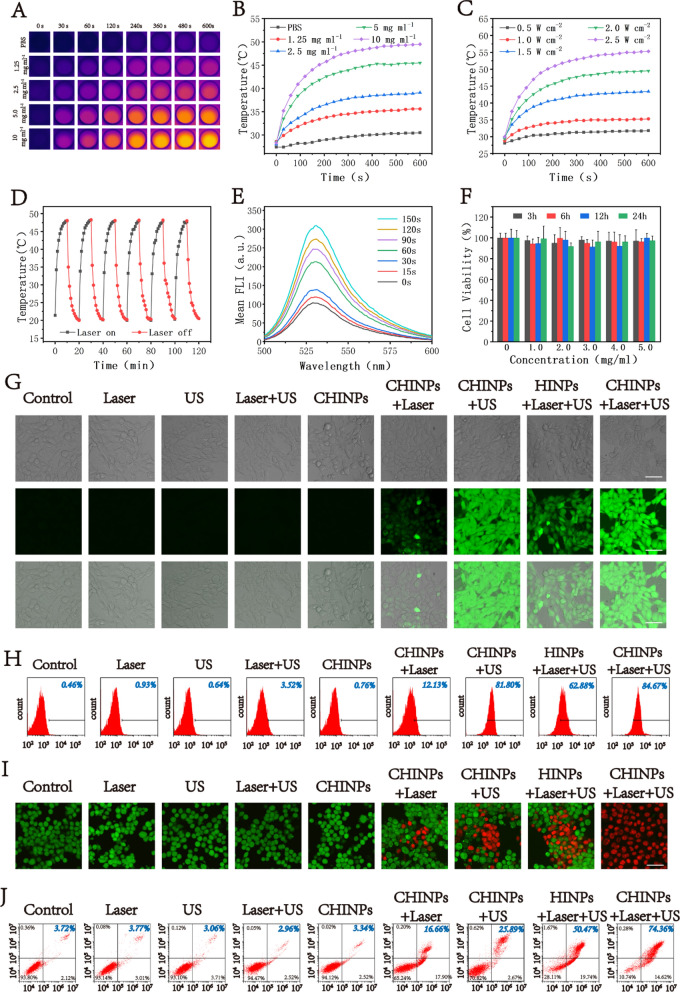
In vitro cytotoxicity and synergistic antitumor effects of PTT/SDT. **A** Infrared thermal images of the CHINPs at different concentrations under 808 nm NIR laser irradiation (2.0 W cm^−2^, 10 min). **B** Photothermal temperature–time curves of the CHINPs at different CHINPs concentrations and **C** different power densities of 808 nm NIR laser. **D** Photothermal temperature–time curves of an CHINPs aqueous solution for six cycles at a power intensity of 2.0 W cm^−2^ under irradiation by 808 nm NIR laser. **E** The ROS production of CHINPs after different time of US irradiation was detected by SOSG. **F** Relative cell viability of 4T1 cells after coincubation with various concentrations of CHINPs for 3 h, 6 h,12 h and 24 h. **G** ROS production in 4T1 cells after different treatments was observed by DCFH-DA staining and CLSM. Scale bar: 50 μm. **H** Flow cytometry of ROS production in 4T1 cells as stained with DCFH-DA after different treatments. **I** CLSM images of 4T1 cells costained with PI (red fluorescence) and calcein-AM (green fluorescence) after different treatments, Scale bar: 50 μm. **J** Flow cytometry apoptosis assay of 4T1 cells after the incubation with CHINPs under different treatments followed by staining with Annexin-FITC and PI

Under the irradiation of 808 nm laser (2 W cm^−2^, 10 min), an obvious photothermal effect was shown with a time and dose dependent manner (Fig. 3A, B). The temperature was elevated with the increasing time and CHINPs concentration and reached as high as 49.5 ℃ at the concentration of 10 mg mL^−1^. As laser intensity increased, the temperature also rose in the presence of CHINPs (Fig. 3C and Additional file 1: Fig. S12). Moreover, no obvious deterioration for the photothermal performance of CHINPs was found during six laser on/off cycles (Fig. 3D), showing the high photothermal stability. This property may be benefitted from the cell membrane coating [57].

HMME in CHINNPs is a promising sonosensitizer and the level of ROS production is crucial for SDT effectiveness [76]. Under the US irradiation, the ROS production in CHINPs was increased over the durations as determined by SOSG probe (Fig. 3E and Additional file 1: Fig. S13). CHINPs had no significant effect on the survival rate of 4T1 cells without US irradiation as shown by CCK-8 assay (Fig. 3F), indicating the absence of ROS.

The intracellular DCFH-DA was converted into 2',7'-dichlorofluorescein (DCF) by ROS, which could be observed for green fluorescence under CLSM. No obvious DCF fluorescence was displayed in cells treated with CHINPs alone, Laser alone, US alone or Laser plus US (Fig. 3G). Weak fluorescence was shown in cells treated with CHINPs plus Laser while strong fluorescence was found in cells in the other three groups. The cells treated with CHINPs plus US and Laser exhibited the strongest DCF fluorescence, indicating that a largest amount of ROS was generated in this group. The cellular ROS levels was further quantitatively analyzed by flow cytometry, which showed the similar trend to the observations under CLSM (Fig. 3H). The robust ROS yield from CHINPs exposed to US irradiation offers a great promise for effective SDT.

To further assess the therapeutic effect of PTT and SDT with CHINPs on 4T1 cells, Calcein-AM/PI staining were used to identify live cells (green fluorescence) and dead cells (red fluorescence). As shown in CLSM images, almost all cells presented red fluorescence after the treatment of CHINPs with Laser plus US irradiation, indicating cell apoptosis/necrosis. Comparatively, cells only treated with Laser, US, Laser plus US or CHINPs alone presented obvious green fluorescence, suggesting no cell death. The treatments of Laser or US combined with CHINPs and Laser plus US with HINPs also caused some cell death (Fig. 3I). The cell damage was further quantitatively assessed by flow cytometry, which was consistent with Calcein-AM/PI staining results. The treatment of PTT plus SDT with CHINPs resulted in the highest cell apoptosis rate (74.36%) (Fig. 3J).

### MRI/PAI performance evaluation of CHINPs

**Fig. 4:**
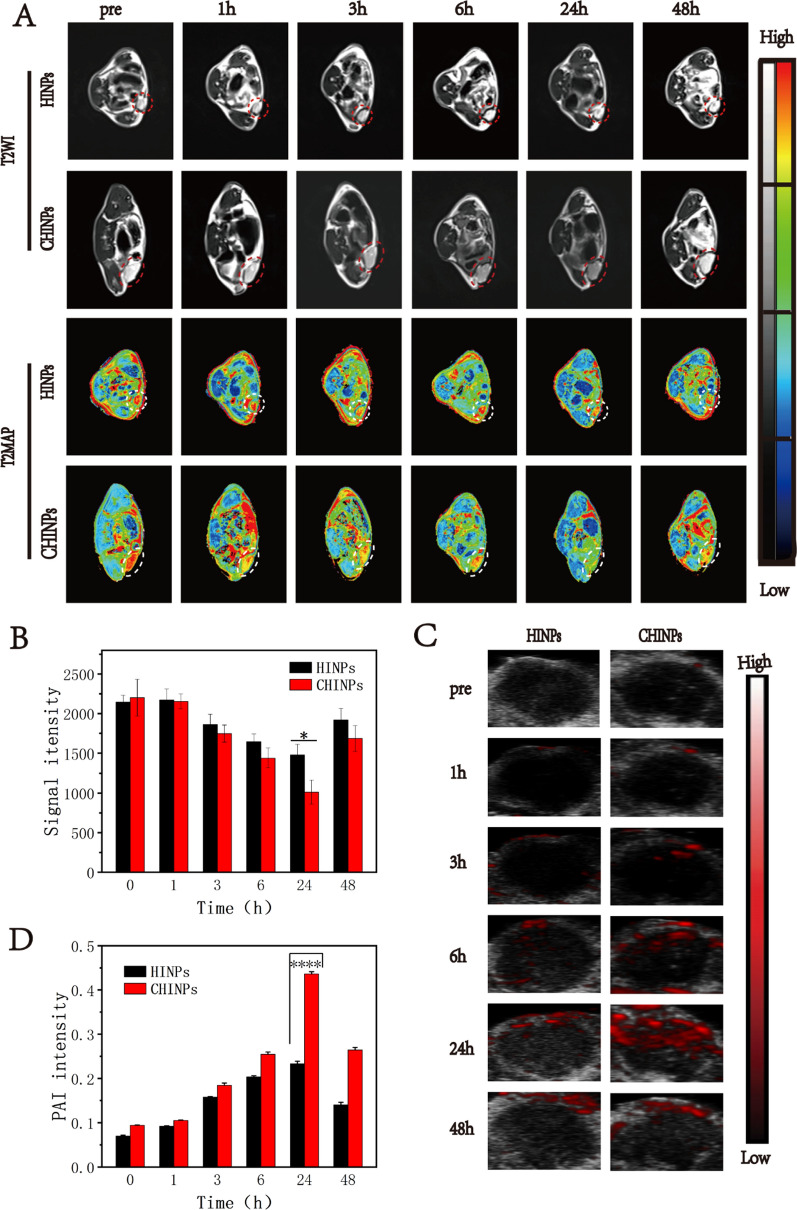
In vivo MRI and PAI performance of CHINPs. **A** T2-weighted MRI and T2-mapping images of 4T1 tumor-bearing mice at different time points after HINPs, CHINPs injection. **B** The corresponding Signal intensity values in the tumor region. **C** In vivo PAI of tumor regions in 4T1 tumor-bearing mice at different time points after HINPs, CHINPs injection and **D** the corresponding signal intensity values. Data shown are mean ± SD (n = 3). Statistical differences determined by one-way ANOVA; *p < 0.05, ****p < 0.0001

As revealed in Additional file 1: Fig. S14, the CHINPs at different concentrations negatively enhanced T2-weighted MRI in vitro. The MRI signal intensity and the relaxation time decreased in accordance to the increase of CHINPs concentration (Additional file 1: Fig. S15). The potential of CHINPs for T1 contrast imaging was also investigated in vitro. As shown in Additional file 1: Fig. S16, CHINPs were still unmapped even their concentration went up to 1.664 mM in T1-weighted imaging. However, CHINPs were mapped at a much lower concentration of 0.026 mM in T2-weighted imaging, indicating that CHINPs were more suitable to act as a T2 contrast agent. Importantly, the intravenous administration of CHINPs or HINPs nanoparticles into 4T1 tumor-bearing mice produced evident negative enhancement in T2-weighted and T2-MAP MR images in tumor region, indicating that successful encapsulation of SPIO into the nanoparticles. CHINPs showed better performance to enhance T2-weighted imaging compared to HINPs based on the quantitative analysis of signal intensity, suggesting homologous targeting effect from cell membrane coating (Fig. 4A, B).

It is noted that both SPIO and HMME can enhance PAI due to their strong light absorption within the range of near infrared wavelength [77]. CHINPs, CINPs and CHNPs showed a wide absorption spectrum over the range of 680 ~ 950 nm excitation wavelengths, and enhanced PA imaging in a concentration dependent manner in vitro (Additional file 1: Figs. S17, 18). It seemed like SPIO had better performance to enhance PA imaging as the PA values of CINPs and CHINPs were significantly higher than that of CHNPs at the same concentrations (Additional file 1: Fig. S19). Furthermore, CHINPs caused stronger enhancement for PAI on 4T1 tumor-bearing mice compared to HINPs, which may be attributed to their high accumulation in tumor region (Fig. 4C, D).

### In vivo photothermal performance

**Fig. 5:**
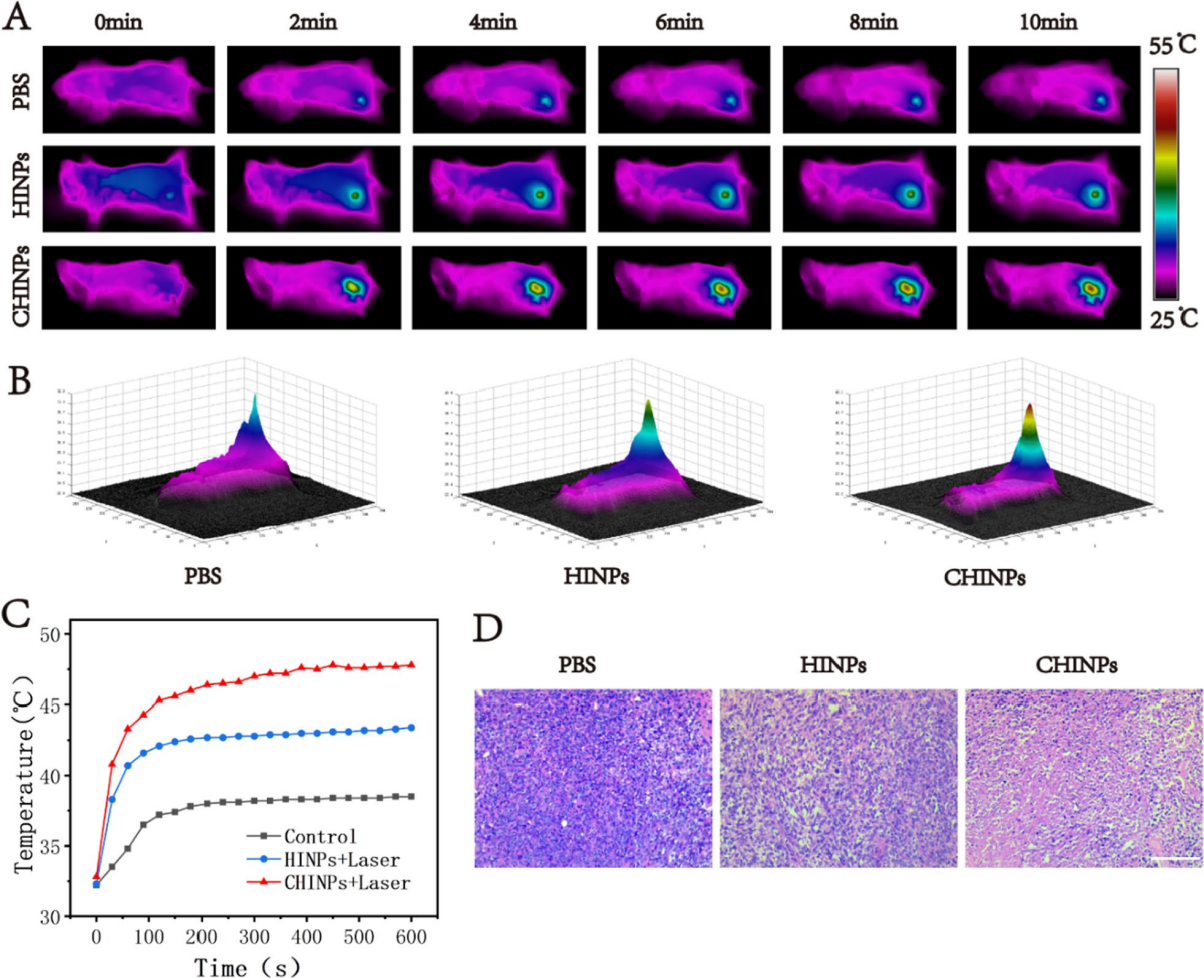
In vivo photothermal performances of the CHINPs. **A** Thermal images of 4T1 tumor-bearing mice of different groups (Laser, HINPs + Laser, CHINPs + Laser) and **B** Corresponding 3D images within tumor regions. **C** Photothermal temperature–time curves of the aforementioned three groups under laser irradiation. **D** H&E-stained images of the tumors in the aforementioned three groups. Scale bar: 100 µm

At 24 h after injection with CHINPs or HINPs, the nanoparticles accumulation peaked in tumor region according to the collective results from IVIS, PAI and MRI, and then PTT were initiated. The temperature in tumor region rapidly increased to 52.9 °C under 808 nm laser irradiation (2.0 W cm^−2^, 10 min) with CHINPs, which resulted in much stronger tumor ablation compared to that with HINPs (Fig. 5A–C). The histopathological results further confirmed that more extensive tumor necrosis was found in CHINPs group compared to that in HINPs group (Fig. 5D).

### Synergy of PTT/SDT with anti-PD-1 for tumor suppression

**Fig. 6:**
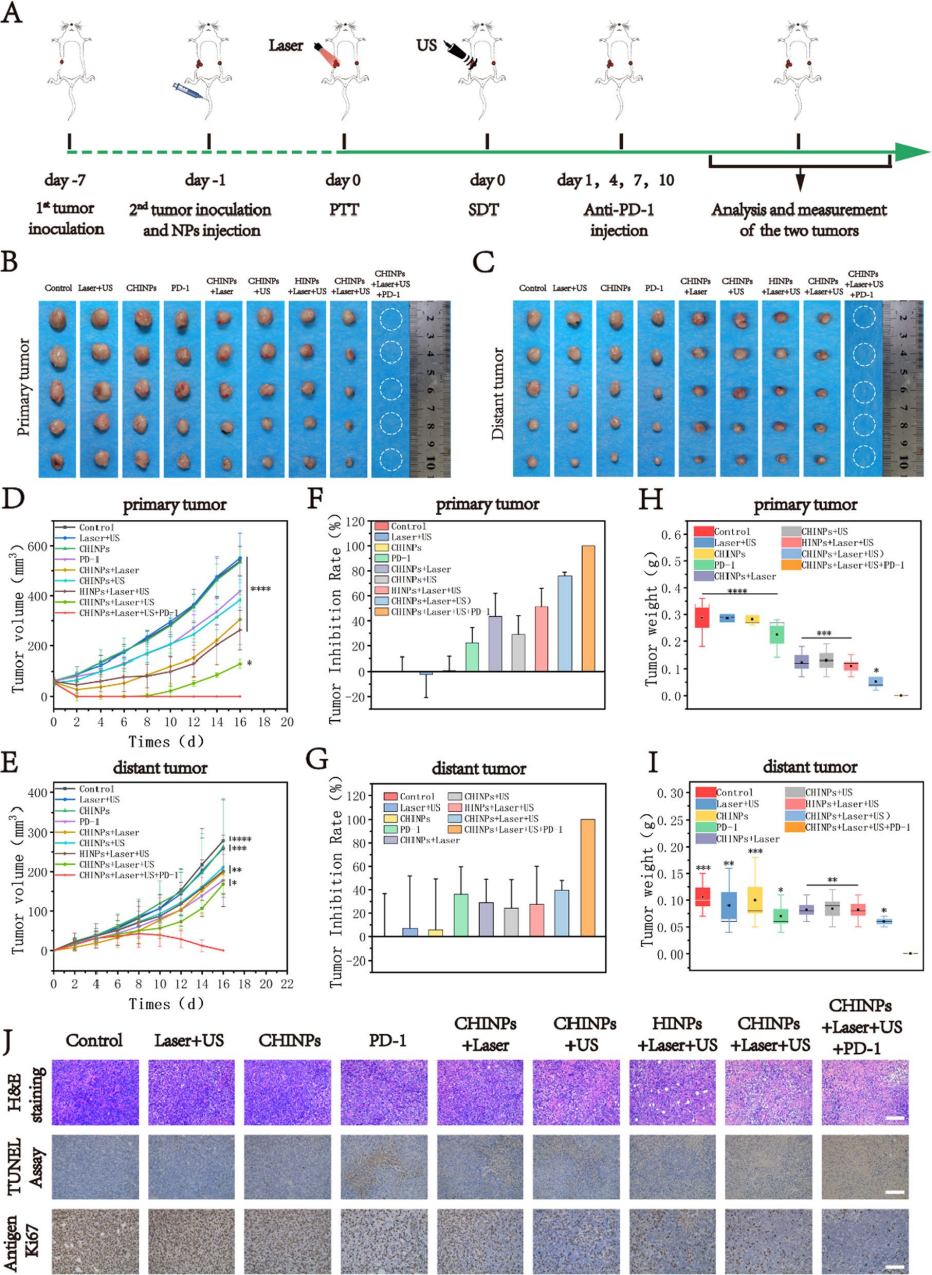
In vivo anticancer activity of PTT/SDT plus anti-PD-1 immunotherapy. **A** Schematic illustration of the in vivo experimental design. Tumor on the right abdomen was designated the primary tumor, and that on the left site was defined as the distant tumor to mimic metastasis. **B**–**I** Photographs of excised primary (**B**) and distant (**C**) tumors of different groups of 4T1 tumor-bearing mice after various treatments; Primary (**D**) and **E** distant tumor-growth curves of different groups; Tumor inhibition rate of primary (**F**) and distant (**G**) tumors at the end of treatments; average weights of primary (**H**) and distant (**I**) tumors at the end of treatments. Data shown are mean ± SD (n = 5), Statistical differences determined by one-way ANOVA. **J** H&E staining, TUNEL staining and Antigen Ki-67 immunohistochemistry staining in tumor region of each group after the treatments. Scale bar: 100 μm. Data shown are mean ± SD (n = 3). Statistical differences determined by one-way ANOVA; *p < 0.05, **p < 0.01, ***p < 0.001, **** < 0.0001

Tumor metastasis is the main cause of cancer-induced deaths [78, 79]. CHINPs-augmented PTT/SDT eliminated the primary tumor and simultaneously induced anti-tumor immune response by producing tumor fragments to release antigens, which facilitated PD-1 blockade to prevent tumor metastasis. The synergistic therapeutic effect was investigated on orthotopic 4T1 bilateral tumor model. The experimental procedure was shown in Fig. 6A, tumor-bearing mice were irradiated with an 808 nm laser for PTT and/or irradiated with US for SDT at 24 h after each i.v. injection of the nanoparticles, respectively. Anti-PD-1 antibodies were intraperitoneally injected into mice at the dose of 50 μg/mouse on the 1st, 4th, 7th and 10th day after irradiation. The results of different treatments against the primary and mimic distant tumors were shown in Fig. 6B–G. No therapeutic effect was observed after treatments with Laser plus US or CHINPs alone. Anti-PD-1, PTT or SDT with CHINPs, PTT plus SDT with HINPs exhibited a slight inhibition effect on primary and distant tumors. Comparatively, the combined PTT and SDT with CHINPs significantly inhibited tumor growth, but the tumor relapsed 7 days later and the distant tumor growth was still not controlled. However, both the primary and distant tumors were completely eliminated when anti-PD-1 therapy was added after the treatment of PTT plus SDT in the presence of CHINPs. Notably, the triple therapy of CHINPs augmented PTT/SDT combined with anti-PD-1 blockade led to synergistic therapeutic efficacy. These findings were further confirmed by the quantitative analysis of tumor weight after treatment as indicated in Fig. 6H and I.

At day 3 after treatment, H&E, TUNEL and Ki67 staining of tumor tissues in one mouse were performed to investigate tumor growth. The combined treatment of PTT and SDT in the presence of CHINPs exhibited significantly higher tumor apoptosis and lower proliferation index compared to other groups (Fig. 6J, Additional file 1: Figs. S20, S21).

### Mechanism of systematic anti-tumor immune response

**Fig. 7:**
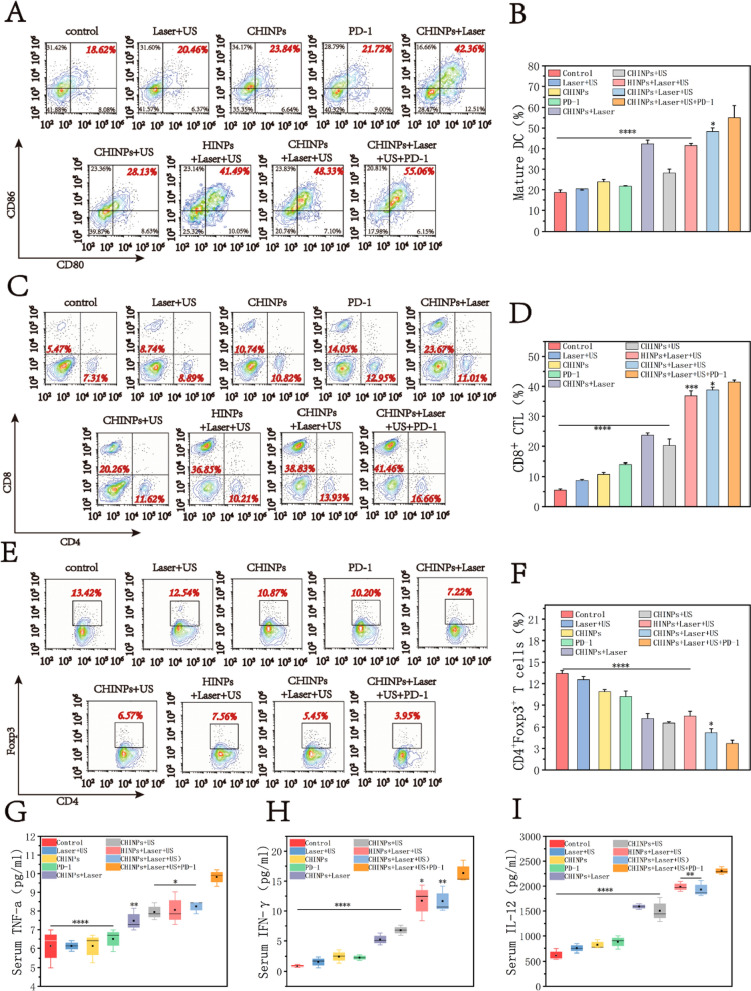
In vivo activation of immune responses triggered by PTT/SDT. **A** The DC maturation levels induced by CHINPs-based PTT/STT on 4T1 tumor-bearing mice (gated on CD11c^+^ DC cells) analyzed by flow cytometry and **B** the corresponding quantitative analysis. **C** The CD8^+^ T cells (CD3^+^CD8^+^) analyzed by flow cytometry and **D** the corresponding quantitative analysis. **E** The Treg cells (CD3^+^CD4^+^Foxp3^+^) analyzed by flow cytometry and **F** the corresponding quantitative analysis. **G**–**I** Cytokine levels in sera from mice isolated at 7d post different treatments. Data shown are mean ± SD (n = 3). Statistical differences determined by one-way ANOVA; *p < 0.05, **p < 0.01, ***p < 0.0001, ****p < 0.0001

The tumor tissue fragments induced by PTT or SDT can release tumor antigens as an in situ vaccine to stimulate DCs maturation [29–34], which are crucial for stimulating an efficient adaptive immune response by activating T lymphocytes [80, 81]. To understand the underlying mechanisms of antitumor effect triggered by CHINPs-augmented PTT/SDT with anti-PD-1(triple therapy), the percentage of mature DCs in the mimic distant tumors were assessed on day 7 after treatment by flow cytometry assay. The results showed that the relatively high percentages of mature DCs was shown in the group of CHINPs with Laser (42.36 ± 1.79%), HINPs with Laser plus US (41.49 ± 1.02%) and CHINPs with Laser plus US (48.33 ± 1.73%), while the triple therapy group resulted in the highest percentage (55.06 ± 5.89%) of mature DCs (Fig. 7A, B). Moreover, the percentage of activated CD8^+^ T and CD4^+^ T cells in tumors were further analyzed. As shown in Fig. 7C, D, the proportion of CD8^+^ T cells in the triple therapy group (41.46 ± 0.68%) was significantly higher than those in other groups, which was consistent with the proportion of mature DCs. Based on the level of Foxp3 marker, CD4^+^ T cells can be classified into two types, i.e., effective T cells (Teffs, CD4+ Foxp3−) and regulatory T cells (Tregs, CD4+ Foxp3+). Tregs hamper the anti-tumor immune response by antagonizing activated immune cells [82]. The percentage of immunosuppressive Tregs in the triple therapy group significantly decreased, which was lower than those in other groups (Fig. 7E, F). These results indicated that the triple therapy strategy can ameliorate tumor immunosuppression microenvironment. Additionally, serum cytokines including IL-12, IFN-γ and TNF-α play vital role in cellular immunity against cancer. They were analyzed by ELISA at day 7 post treatment. Similar to the results of mature DCs analysis, the levels of these cytokines were remarkable boosted in mice received triple therapy, indicating the establishment of anti-tumor immune response (Fig. 7G–I).

Taken together, these findings clearly suggest that the triple therapy strategy of CHINPs augmented PTT/SDT with anti-PD-1 blockade not only destroys the primary tumor, but simultaneously activates systemic anti-tumor immune response to eliminate distant tumor through promoting DCs maturation, activating CD8^+^ T cells, alleviating the immunosuppressive tumor microenvironment and increasing the expression of serum cytokines.

### In vivo biosafety of CHINPs

The in vivo acute toxicity and long-term toxicity of CHINPs were evaluated in healthy Kunming mice, respectively. As shown in Additional file 1: Fig. S22, all the hematologic and serum biomedical indexes of mice were maintained at normal levels at day 1, 7, 14, 28 after i.v. administration of CHINPs, indicating their good biosafety in vivo. Furthermore, the main organs (heart, liver, spleen, lung and kidney) were collected and sectioned for H&E staining. As shown in Additional file 1: Fig. S23, no distinct histomorphology change was found at different days.

## Conclusion

In summary, in this study, we report a rational combined strategy with PTT, SDT and PD-1 blockade immunotherapy to inhibit tumor growth and metastasis. HMME and SPIO as sonosensitizers and PTAs were loaded into CCMs modified PLGA nanoparticles to fabricate CHINPs as a novel biomimetic nanoprobe. The CHINPs accumulated in tumor region benefiting from their homologous targeting ability and then augmented SDT and PTT effect. Moreover, SDT overcame the inherent deficiency of PTT in targeting deeper tumors and PTT enhanced SDT by increasing blood flow and oxygenation of the tumors. Therefore, CHINPs-augmented photothermal-sonodynamic combined therapy generated favorable synergistic antitumor effect. Furthermore, systematic immune responses induced by PTT and SDT, including elevation of CD8^+^ T cells and decrease of Tregs, as well as the enhanced DC maturation and cytokine secretion, have been shown to be responsible for the enhanced immunotherapy and inhibited metastasis. Lastly, CHINPs as a multimodal nanoprobe simultaneously enhanced MR/PA/PI imaging, providing imaging guidance for precise tumor therapy. Given the above, a triple-combined therapeutic modality was established by the integration of biomimetic nanoprobe-based SDT/PTT with anti-PD-1 immunotherapy for eliminating primary and metastatic tumors.

## Supplementary Information

**Additional file 1: **Additional figures.

## Data Availability

All data generated or analysed during this study are included in this published article [and its additional files].

## Abbreviations

PTT: Photothermal therapy
SDT: Sonodynamic therapy
SPIO: Superparamagnetic iron oxide
HMME: Hematoporphyrin monomethyl ether
PTAs: Photothermal transduction agents
NIR: Near infrared
ROS: Reactive oxygen species
TME: Tumor microenvironment
RES: Reticuloendothelial system
PLGA: Polylactic glycolic acid
CCMs: Cancer cell membranes
PAI: Photoacoustic imaging
MRI: Magnetic resonance imaging
PTI: Photothermal imaging
IVIS: Living fluorescence imaging system
ICP-MS: Inductively coupled plasma mass spectrometry
LIFU: Low-intensity focused ultrasound
DCs: Dendritic cells
EE: Encapsulation efficiency
LC: Loading capacity
SOSG: Singlet Oxygen Sensor Green
DCFH-DA: 2',7'-Dichlorofluorescein diacetate
DIO: 3,3′-Dioctadecyloxacarbocyanine perchlorate
