# Supplementary material for: Biomimetic nanoprobe-augmented triple therapy with photothermal, sonodynamic and checkpoint blockade inhibits tumor growth and metastasis
Source: J Nanobiotechnology. 2022 Feb 15;20:80. doi: 10.1186/s12951-022-01287-y (PMC8845357; doi:10.1186/s12951-022-01287-y)
Supplement: Supplementary file 1 — Additional file 1: Additional figures. [file 12951_2022_1287_MOESM1_ESM.docx]

**Supporting information**

**Biomimetic Nanoprobe-augmented Triple Therapy with Photothermal, Sonodynamic and Checkpoint blockade Inhibits Tumor Growth and Metastasis**

*Xiaohong Lin^1#^, Tao He^2#^, Rui Tang^1#^, Qianru Li^3^, Nianhong Wu^1^, Yin Zhou^1^, Hongye He^1^, Li Wan^1^, Ju Huang^3^, Qinqin Jiang^1^, Yixin Zhong^3^, Zhuoyan xie^1^, Zhongqian Hu^4^, Yang Zhou ^5^, Pan Li^1^**

**Supplementary figures**





**Figure S1.** Corresponding loading capacity of different doses of HMME. Data shown are mean ± SD (n = 3).





**Figure S2.** Corresponding the encapsulation rate of different doses of HMME. Data shown are mean ± SD (n = 3).





**Figure S3.** UV-Vis spectrum of CHINPs prepared with different doses of HMME.





**Figure S4.** Corresponding loading capacity of different doses of SPIO. Data shown are mean ± SD (n = 3).





**Figure S5.** Corresponding the encapsulation rate of different doses of SPIO. Data shown are mean ± SD (n = 3).


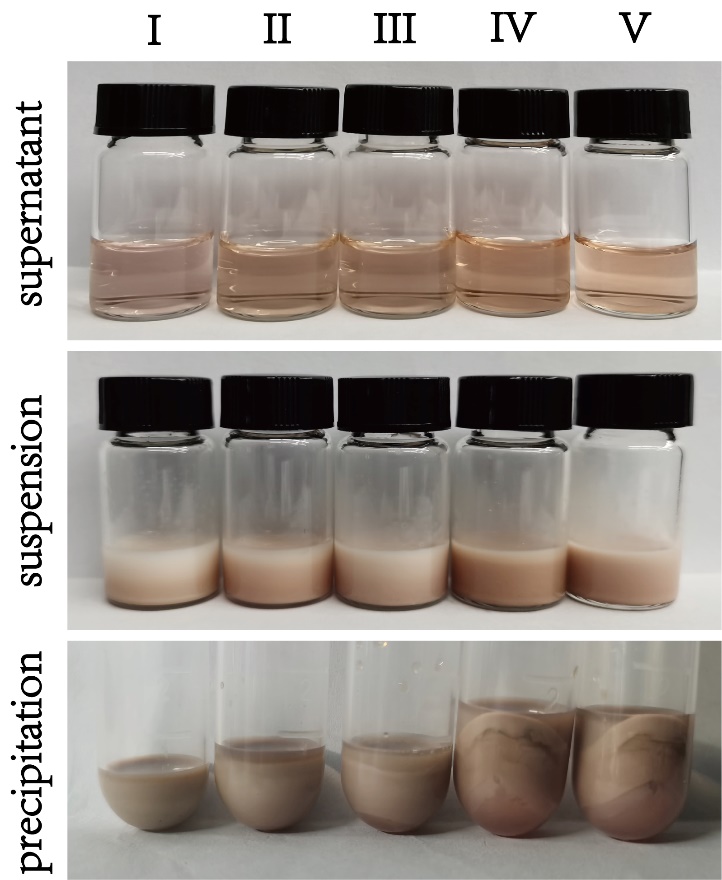


**Figure S6.** Photographs of HINPs prepared with different doses of HMME and SPIO. (HMME: I:0.8mg, II:1.0mg, III:1.2mg, IV:1.4mg, V:1.6mg; SPIO: I:0.6mg, II:0.75mg, III:0.9mg, IV:1.05mg, V:1.2mg.)


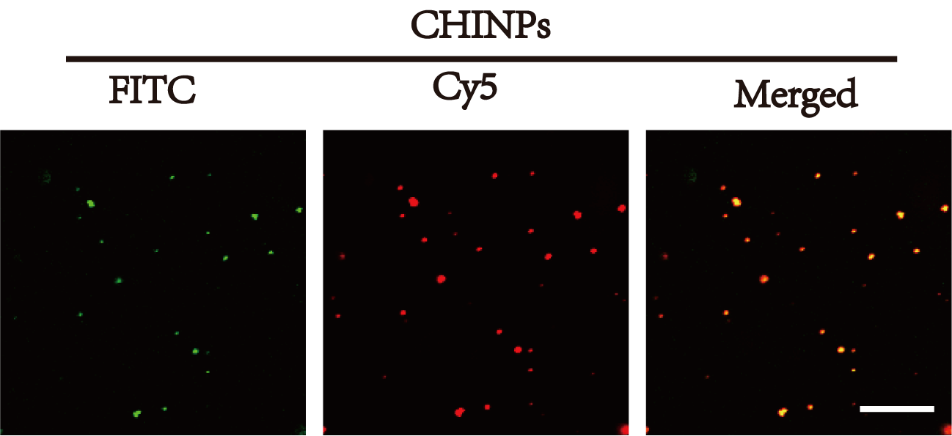


**Figure S7.** Laser confocal microscopy image of HINPs(red) coated by cell membrane(green). Scale bar: 25μm.


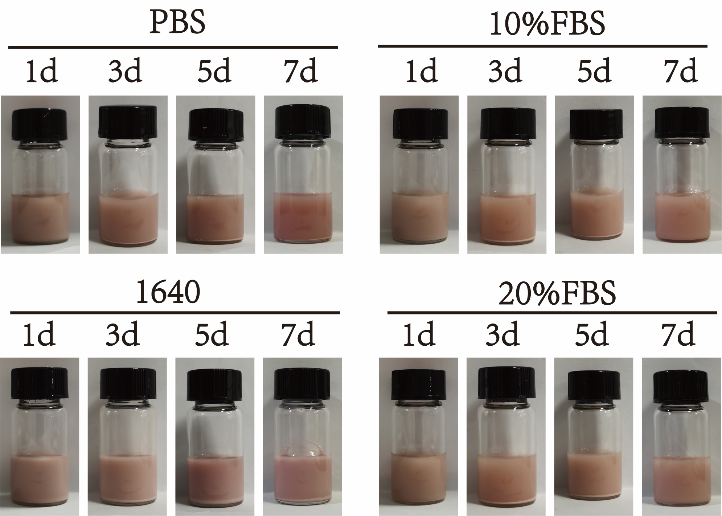


**Figure S8.** Photographs of stability of CHINPs in PBS, water containing 1640 medium, 10% or 20% FBS.


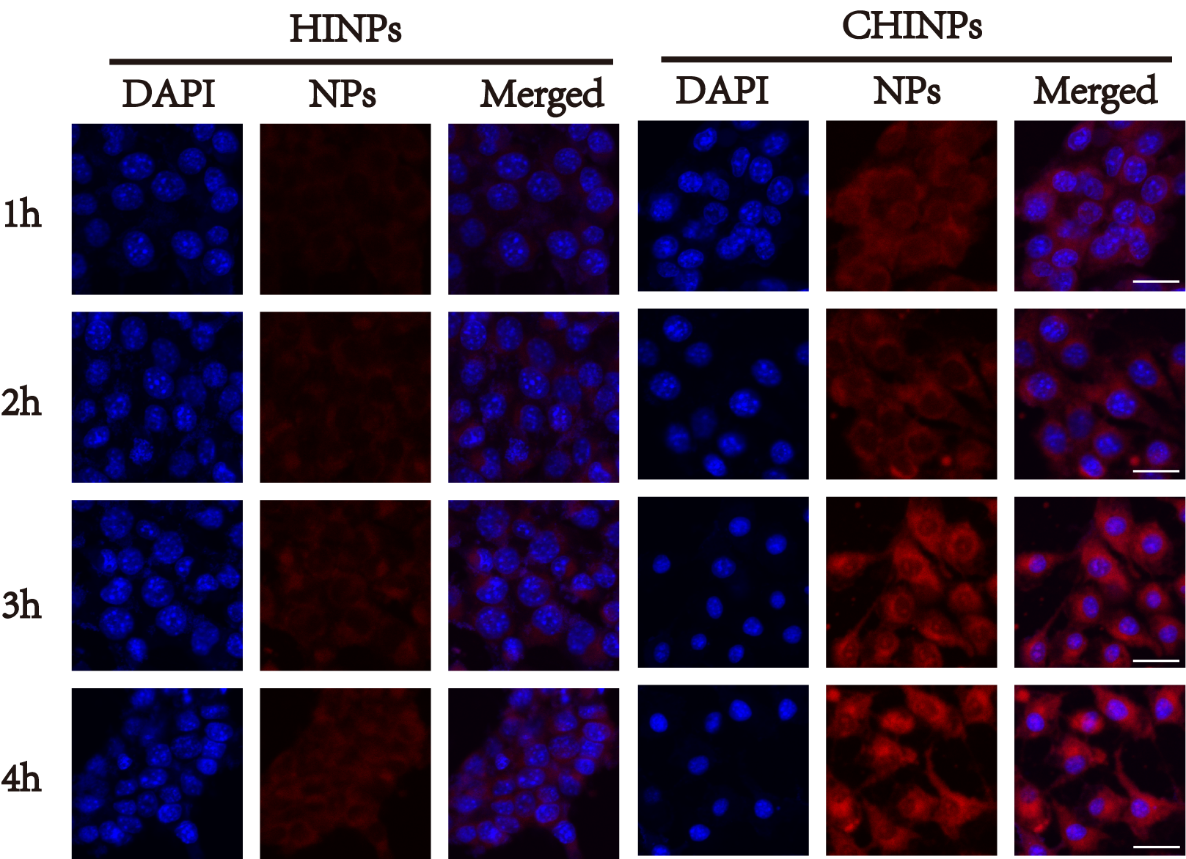


**Figure S9.** CLSM images of 4T1 cells treated with HINPs and CHINPs. Scale bar: 20μm.


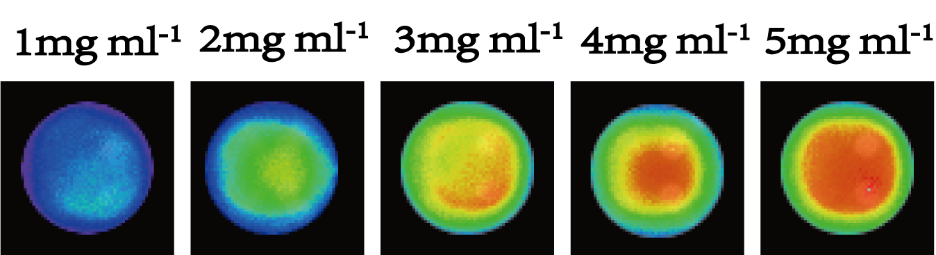


**Figure S10.** fluorescence imaging of the CHINPs at different concentrations.


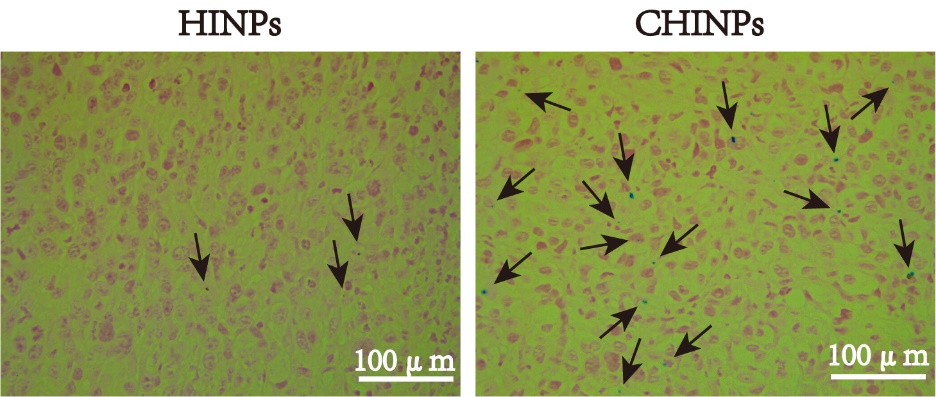


**Figure S11.** Prussian blue staning of tumor slices after adiministration of HINPs and CHINPs for 24 h. Scale bar:100μm.


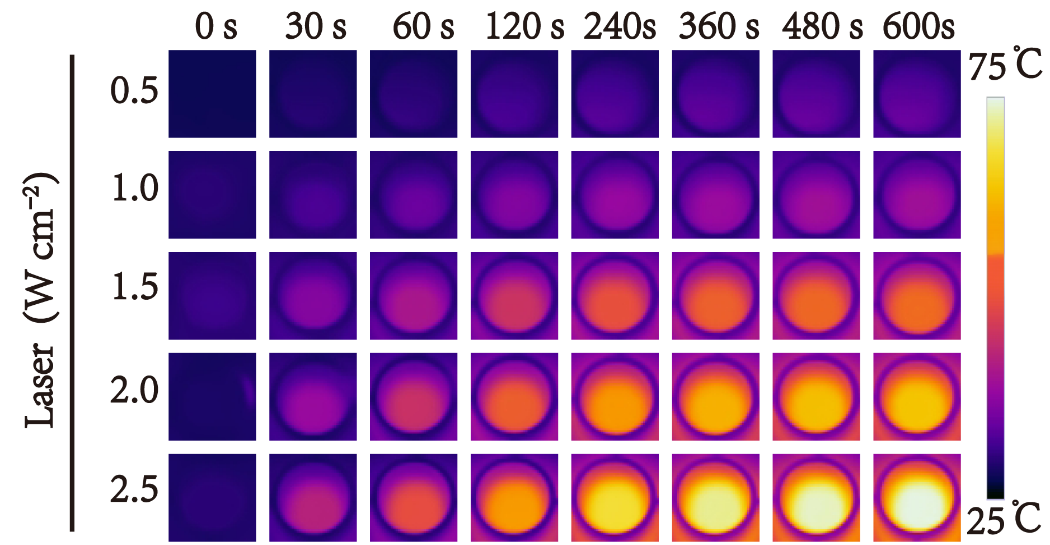


**Figure S12.** Infrared thermal images of CHINPs under 808 nm laser irradiation with different

power density.





**Figure S13.** The ROS production of CHINPS under different irradiation time was detected by SOSG.


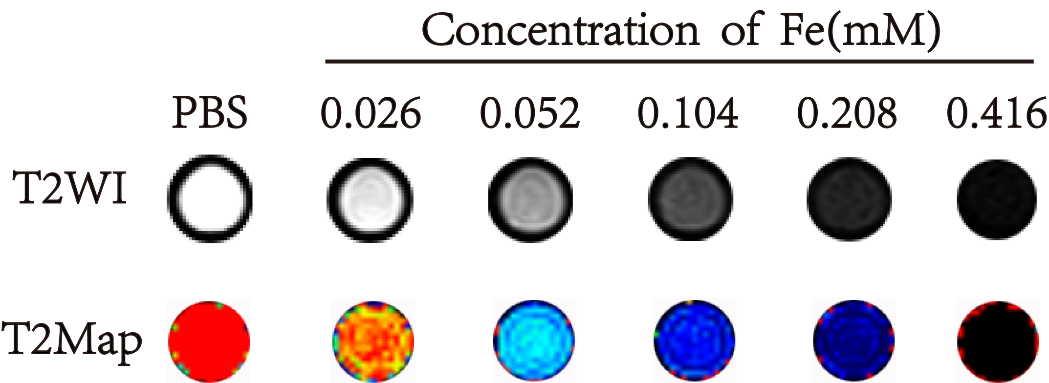


**Figure S14.** T2-weighted MR images and T2-mapping images of the CHINPs at different concentrations.





**Figure S15.** Corresponding T2 relaxation rate of the CHINPs at different concentrations.


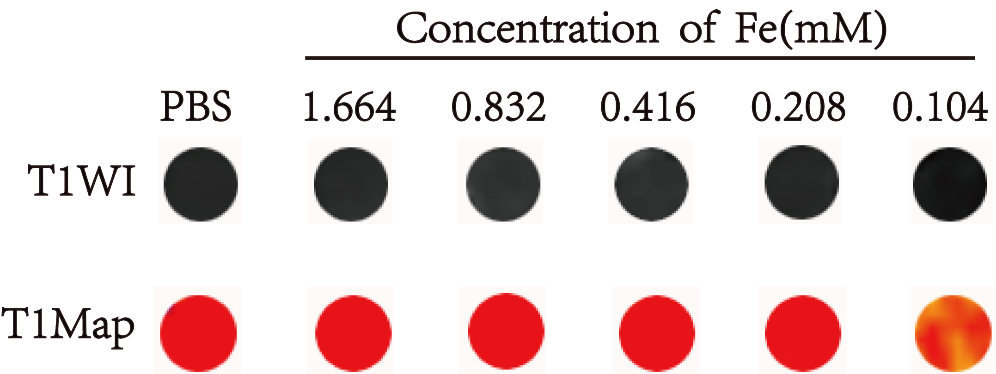


**Figure S16.** T1-weighted MRI and T1-mapping images of the CHINPs at different concentrations.





**Figure S17.** PAI intensity of CHNPs, CINPs and CHINPs under full-spectrum scanning (ranging from 680 to 950 nm).


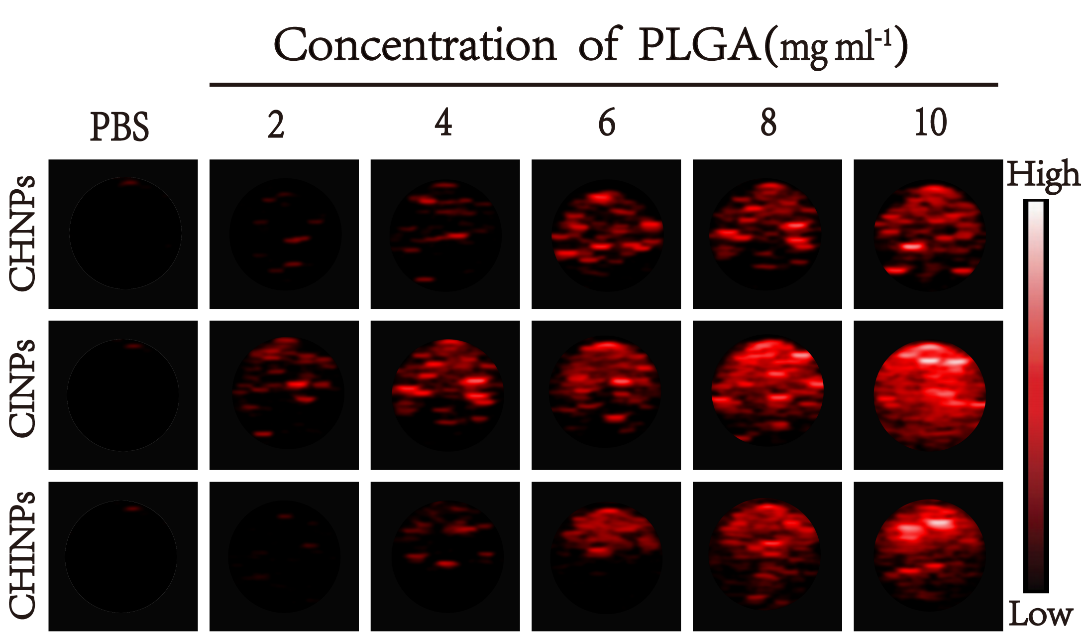


**Figure S18.** PAI in PA-mode of the CHNPs, CINPs and CHINPs at different concentrations.





**Figure S19.** Corresponding intensities of PA signal of the CHNPs, CINPs and CHINPs at different concentrations.





**Figure S20.** Quantitative analysis of the proliferation index in different groups. Data shown are mean ± SD (n = 3). Statistical differences determined by one-way ANOVA; *p < 0.05, **p < 0.01, ***p < 0.001, ****<0.0001.





**Figure S21.** Quantitative analysis of the apoptotic index in different groups. Data shown are mean ± SD (n = 3). Statistical differences determined by one-way ANOVA; *p < 0.05, **p < 0.01, ***p < 0.001, ****<0.0001.


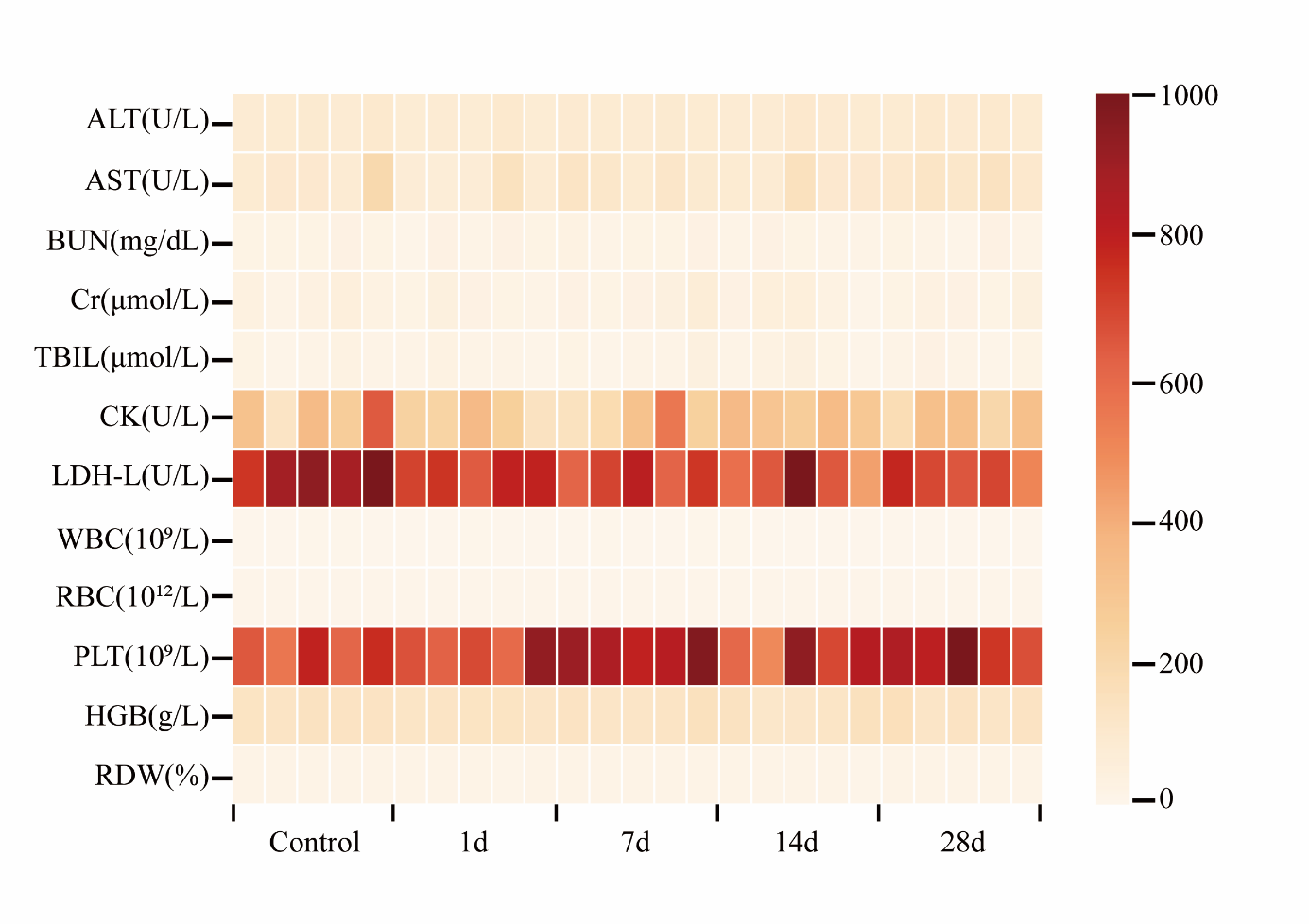


**Figure S22.** The hematological and blood biochemical test of mice after i.v. administration of CHINPs at different time points.


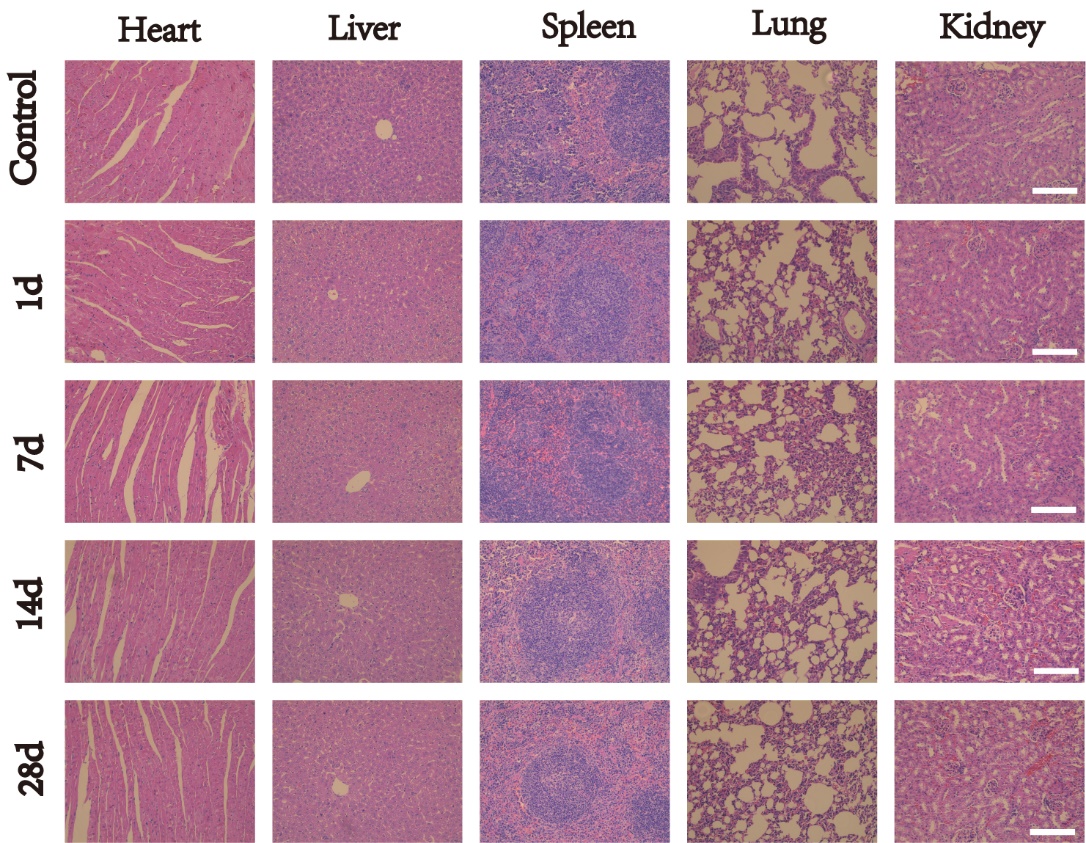


**Figure S23.** H&E staining of the major organs including heart, liver, spleen, lungs and kidneys after the mice were sacrificed at 1d, 7d, 14d and 28d post intravenous injection with CHINPs. Scale bar: 100μm.
